# The role of human papillomavirus in oral squamous cell and verrucous carcinomas: a systematic review with case series

**DOI:** 10.3389/or.2026.1798220

**Published:** 2026-04-22

**Authors:** Luisa Limongelli, Alessio Danilo Inchingolo, Grazia Marinelli, Francesco Inchingolo, Gianfranco Favia, Lucia Casamassima, Irma Trilli, Laura Ferrante, Marta Forte, Angelo Michele Inchingolo, Gianna Dipalma

**Affiliations:** 1 Department of Interdisciplinary Medicine, University of Bari “Aldo Moro”, Bari, Italy; 2 Department of Biomedical, Surgical and Dental Sciences, Milan University, Milan, Italy

**Keywords:** HPV, human papillomavirus, oral cancer, oral squamous cell carcinoma, verrucous carcinoma

## Abstract

**Introduction:**

Oral cancer (OC), particularly squamous cell carcinoma and its variants such as verrucous carcinoma, represents a growing public health concern due to increasing global incidence. While tobacco and alcohol remain the main risk factors, attention has turned to the potential role of human papillomavirus (HPV), particularly with particular consideration on high-risk genotypes.

**Objective:**

This systematic review evaluates current evidence on the association between HPV and OC.

**Methods:**

A structured search was conducted in PubMed, Scopus, and Web of Science using keywords including “oral carcinoma,” “oral squamous cell carcinoma,” “oral verrucous carcinoma,” and “HPV.” Screening followed PRISMA guidelines, and 15 articles were selected. Additionally, a case series of patients treated at the Department of Interdisciplinary Medicine, University of Bari “Aldo Moro,” are presented to provide clinical context. Results. The evidence suggests a possible association between HPV infection, especially genotype 16, and a subset of oral squamous cell carcinomas. However, differences in detection techniques and study design contribute to variability in findings.

**Conclusion:**

While HPV may play a role in oral carcinogenesis, further high-quality studies are required to clarify its impact. These findings may have implications for screening, prognosis, and prevention strategies, including HPV vaccination.

## 1 Introduction

### 1.1 General background

Oral cancer (OC), especially oral squamous cell carcinoma (OSCC) and its variants like verrucous carcinoma (OVC), poses a significant global health challenge, with increasing incidence rates reported in both developed and developing nations (1, 2). Despite progress in diagnostic techniques and multidisciplinary treatments, OC prognosis remains poor, with 5-year survival rates stuck around 50%–60% (3–10). The latter highlights the need for a deeper understanding of its causes and biological mechanisms to enhance prevention, early detection, and targeted therapies (11–14).

### 1.2 Risk factors and HPV oncogenesis

**FIGURE 1:**
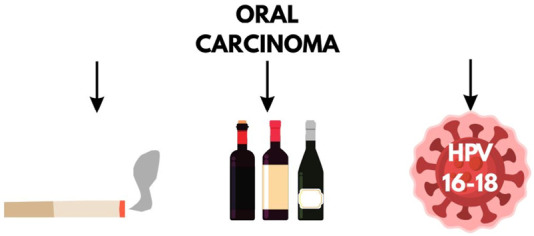
Schematic representation of the main risk factors for OC. Tobacco use, alcohol consumption, high-risk and HPV genotypes (16 and 18) are shown as key contributors to the development of OC.

Historically, the main risk factors for OC have been tobacco use and alcohol consumption, which work together to promote carcinogenesis (Figure 1) (15–22). However, recent evidence has highlighted the role of human papillomavirus (HPV) as a potential additional cause (23–25). HPV is a DNA virus with over 200 known genotypes, with high-risk types such as HPV16 and HPV18 recognized as strong oncogenic drivers (26–32). The oncogenic mechanism of HPV is mainly mediated by the persistent expression of the viral oncoproteins E6 and E7 (33–37). E6 promotes the degradation of the tumor suppressor p53, thereby impairing DNA repair and apoptosis, while E7 inactivates the retinoblastoma protein (pRb), leading to the release of E2F transcription factors and uncontrolled progression through the cell cycle (38–43). The combined loss of these two critical tumor suppressor pathways results in genomic instability, sustained cellular proliferation, and ultimately malignant transformation (44–50).

### 1.3 HPV and head and neck cancers

The causal relationship between HPV and cancers of the uterine cervix is well established, and growing evidence correlates HPV infection to a subset of head and neck squamous cell carcinomas (HNSCC), particularly in the oropharynx (51–57). HPV-positive oropharyngeal cancers are associated with distinct molecular profiles, improved treatment response, and better overall survival compared to HPV-negative tumors (58–64). This has prompted the inclusion of HPV status as a prognostic factor in staging systems and therapeutic decision-making (65–71).

According to the fifth Edition of the WHO Classification of Head and Neck Tumours (2022), both OSCC and OVC are not currently recognized as HPV-related entities. In contrast, HPV-associated oropharyngeal squamous cell carcinoma (OPSCC) represents a distinct clinicopathological entity characterized by non-keratinizing morphology and a strong association with transcriptionally active high-risk HPV infection (72). The diagnosis of HPV-associated OPSCC is based primarily on p16 immunohistochemistry, which serves as a reliable surrogate marker for oncogenic HPV activity. p16 positivity is defined as diffuse nuclear and cytoplasmic staining in ≥70% of tumor cells with moderate to strong intensity. Direct HPV detection assays, such as DNA or RNA *in situ* hybridization and polymerase chain reaction (PCR), may be considered in selected situations—for instance, when p16 staining is equivocal, when morphological features do not match HPV-related patterns, or when required by clinical or research protocols (73, 74).

These diagnostic criteria are essential to distinguish true HPV-driven tumors from those in which viral DNA may merely represent incidental infection.

### 1.4 Controversy of HPV in oral carcinogenesis

By contrast, the role of HPV in oral cavity carcinogenesis is still controversial (75–81). Several studies have reported the presence of HPV DNA, p16 overexpression, or E6/E7 mRNA transcripts in OSCC and OVC, suggesting that HPV may contribute to tumor initiation or progression (82–87). However, other investigations have found little or no evidence of HPV involvement in OC, with prevalence rates varying widely across geographic regions and depending on the detection methods used (88–92). These inconsistencies have generated ongoing debate about whether oral HPV infection is a true causative factor, a passenger infection, or a marker of other underlying processes (93–95).

### 1.5 Clinical and public health implications

Clarifying the role of HPV in OC carries important clinical and public health implications (96–101). If a significant causal association is confirmed, HPV vaccination programs might offer a preventive benefit beyond cervical and oropharyngeal cancers, potentially reducing the incidence of OSCC (102–105). Furthermore, HPV-positive oral tumors may have specific prognostic features that could influence the management, as already observed in HPV-related oropharyngeal cancers (106–109).

### 1.6 Oral VC

OVC is a rare, well-differentiated, low-grade variant of squamous cell carcinoma, first described by Ackerman in 1948 (110–116). It accounts for 0.5%–16% of OSCC and typically arises in the buccal mucosa, gingiva, and tongue, often consisting of abroad-based, outward-growing lesion with a rough, cauliflower-like surface. Such characteristic is histologically confirmed by its exophytic growing instead of a marked invasiveness which may help in differentially diagnose it from OSCC (117–121). Such a characteristic is histologically confirmed by its exophytic growth instead of a marked invasiveness which may help in differentially diagnose it from OSCC. Also, histological markers include a thick, hyperkeratotic squamous epithelium with minimal cytological atypia, prominent keratin plugs, and elongated rete ridges extending into the underlying stroma without true invasion of the basement membrane (122–126). Mitotic activity is generally low, and nuclear pleomorphism is scarce (127–133). Unlike OSCC, which shows infiltrative growth, marked nuclear atypia, higher mitotic activity, and a greater tendency for lymph node or distant metastasis, OVC usually demonstrates a pushing growth pattern, rare metastatic potential, and a more favorable short-term prognosis (134–138). While OVC rarely metastasizes, it can be locally aggressive and tends to recur if not completely excised (121, 139). In some cases, hybrid tumors containing foci of invasive squamous cell carcinoma may occur, conferring a worse prognosis (140–143). These histopathological characteristics are crucial for differential diagnosis, particularly in distinguishing OVC from benign verrucous hyperplasia, proliferative leukoplakia, and conventional SCC (144–147). Also, according to the most recent AJCC classification (2017), the main distinguishing feature in growth pattern is established by the parameter of depth of invasion (DOI), which plays a critical role in staging and prognosis establishment. A schematic representation of the histological features of OVC is presented below. The aim of this systematic review is therefore to critically evaluate and synthesize current evidence regarding the association between HPV and OC, with a focus on squamous cell and verrucous variants (148, 149). By analyzing the available literature, this work seeks to highlight both areas of consensus and points of controversy, providing a clearer perspective on the significance of HPV in oral carcinogenesis and its implications for prevention, prognosis, and treatment (150, 151).

## 2 Materials and methods

### 2.1 Protocol and registration

The current systematic review was conducted following the PRISMA guidelines (Preferred Reporting Items for SR and Meta-Analyses) and International Prospective Register of SR Registry procedures (ID PROSPERO: 1136400).

### 2.2 Search process

The following databases were combed from June 2015 to July 2025, to search for articles published over the last 10 years: PubMed, Web of Science (WoS), and Scopus. The search strategy was developed by combining terms relevant to the study’s purpose. In the advanced search strings used in the databases (detailed search terms are given in Appendix A), the following keywords were applied using Boolean operators to combine terms pertinent to this study’s purpose (Table 1).

**TABLE 1:** Indicators for database searches.

Article-screening strategy	Keywords: “oral carcinoma; oral squamous cell carcinoma; squamous cell oral carcinoma; oral verrucous carcinoma; HPV in oral carcinoma; human papillomavirus; HPV”
	Boolean indicators: OR and AND
	Timespan: June 2015 to July 2025
	Electronic databases: PubMed; Scopus; WOS.

### 2.3 Inclusion and exclusion criteria

The reviewers worked in groups to assess all relevant studies that evaluated:

- Open-access studies written in English
- Full-text articles accessible for review
- Studies conducted *in vivo* on human subjects aged 18 years or older
- Studies involving adults with a diagnosis of OSC or OCV
- Study laboratory-based on human samples,
- Studies with observational design (cross-sectional, case-control, cohort)
- Studies investigating the prevalence, etiological role, prognostic value, or clinicopathological associations of HPV in OSCC and/or OVC

Studies that fulfilled at least one of the following criteria were excluded:

- Systematic reviews, meta-analyses, case reports, or case series
- Letters to the editor, conference abstracts, or commentaries
- Animal model studies
- Exclusively *In vitro* studies
- Studies focusing exclusively on non-oral sites (e.g., oropharynx, larynx) without separate analysis for the oral cavity

### 2.4 PICO question

The PICO format is a framework used in qualitative research to structure clinical research questions. In this study, the PICO addressed the following question: “In adults diagnosed with OSCC compared with those diagnosed with oral verrucous carcinoma, are there differences in the prevalence and etiological role of HPV infection, as well as in associated clinicopathological characteristics and prognosis?? The PICO question was answered as follows:

P (Population)

Adults diagnosed with oral squamous cell carcinoma or oral verrucous carcinoma.

I (Intervention)

Presence of HPV infection (HPV DNA, p16 IHC, ISH, or E6/E7 mRNA).

C (Comparison)

Absence of HPV infection.

O (Outcome)

Differences in HPV prevalence, etiological involvement, and clinicopathological/prognostic characteristics in OSCC and OVC.

### 2.5 Data processing

Five independent reviewers (L.C., I.T., L.F., G.M.,M.F. and F.I.) assessed the methodological quality and risk of bias of the included studies using the ROBINS-I. The tool evaluates key domains such as selection, measurement validity, confounding, and data analysis. Discrepancies in scoring were resolved through discussion and consensus, with support from additional reviewers (L.L., A.D.I., F.I., G.F., A.M.I., and G.D.) when needed. The reviewers screened all retrieved records based on predefined inclusion and exclusion criteria. After screening, a total of 611 articles were imported into Zotero (version 6.0.36) for organization and full-text analysis.

## 3 Results

### 3.1 Selected studies and their characteristics

**FIGURE 2:**
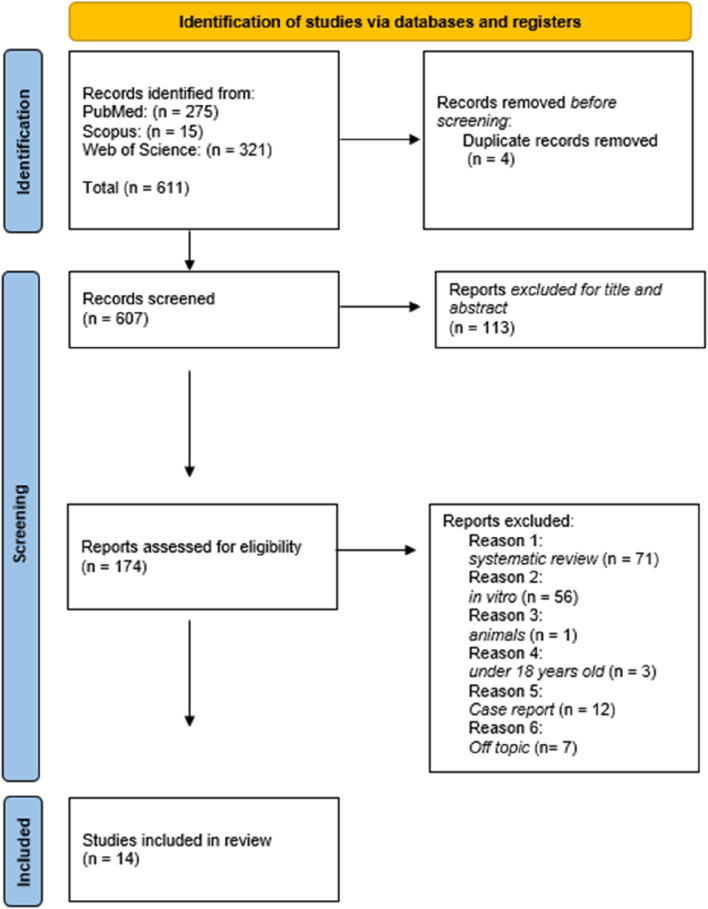
PRISMA flow diagram illustrating the study selection process for systematic review. The chart details the number of records identified, screened, assessed for eligibility, and included in the final analysis, along with reasons for exclusion at each stage.

This PRISMA (Preferred Reporting Items for SR and Meta-Analyses) flow diagram (Figure 2) outlines the rigorous and systematic process used to select studies for the final review. The systematic review process was conducted according to the PRISMA guidelines. A comprehensive search across PubMed (n = 275), Scopus (n = 15), and Web of Science (n = 321) identified a total of 611 records. After the removal of 4 duplicates, 607 articles were screened by title and abstract, leading to the exclusion of 113 records. A total of 174 full-text articles were evaluated for eligibility. Of these, 159 were excluded for the following reasons: systematic review (n = 71), *in vitro* study (n = 56), animal study (n = 1), participants under 18 years old (n = 3), case report (n = 12), and off-topic articles (n = 7). Ultimately, 14 studies met all inclusion criteria and were included in the systematic review (Table 2).

**TABLE 2:** Summary table of included studies.

Authors, year	Country/Setting	Study design	Aim/Objective	Materials and methods	Results	Conclusions
Sgaramella et al., 2015 (152)	Italy	Retrospective cohort	Assess HPV16, p16, syndecan-1 in tongue SCC	109 TSCC cases; ISH for HPV16; IHC for p16, syndecan-1	HPV16 absent; p16+ in 33%; syndecan-1 widely expressed; p16 loss linked to worse prognosis in young patients	HPV16 not involved in TSCC; p16 not surrogate for HPV but prognostic
Chen et al., 2016 (153)	China	Case-control	Investigate HPV16/18 in OSCC and OPMD	99 patients; PCR and sequencing	No HPV16/18 detected; false positives excluded	HPV uncommon in OSCC/OPMD; PCR + sequencing reliable for detection
de Abreu et al., 2018 (154)	Brazil	Cohort study	Assess HPV prevalence in oral cavity SCC	101 patients; PCR with consensus primers; sequencing	HPV detected in 3/90 (3.3%), all HPV16; no associations with clinicopathological features	HPV not implicated in oral cavity SCC in Brazil
Nopmaneepaisarn et al., 2019 (155)	Thailand	Retrospective	Determine HPV prevalence in HNSCC	504 cases; p16 IHC + ISH ± PCR	HPV in 14.5% OPSCC; 1.5% OSCC/LSCC; HPV + linked to better OPSCC survival	HPV rare outside OPSCC; p16 unreliable; regional differences key
Tokuzen et al., 2021 (156)	Japan	Retrospective cohort	Evaluate HPV16 and p16 in OSCC	100 OSCC; RT-qPCR for HPV16 E6 mRNA; IHC for p16	HPV16 mRNA in 1 case (1%); p16+ in 10%; poor concordance	HPV16 rare in OSCC Japan; p16 unreliable surrogate
Li et al., 2021 (157)	China	Retrospective cohort	Assess prognostic role of nodal status by p16	674 OSCC surgical pts; survival analysis	p16+ in 12.6%; prognosis worsened with more positive nodes regardless of p16	Nodal burden is strong prognostic factor, independent of p16
Arsa et al., 2021 (30)	Thailand	Retrospective	Assess p16, HPV, outcomes in HNSCC	662 pts; p16 IHC; PCR subset	p16 in 10.9%; HPV DNA in 4%; p16+ linked to better OS but poor HPV concordance	p16 prognostic but poor HPV surrogate in low-prevalence regions
Sri et al., 2021 (158)	India	Comparative study	Prevalence HPV16/18 in OSCC and PMD	40 archival samples; PCR	HPV16 in 35% OSCC, 5% PMD; no HPV18 detected	HPV16 may play oncogenic role esp. in well-diff. OSCC
Silveira et al., 2022 (159)	Brazil	Cohort study	HR/LR HPV prevalence and prognosis in OSCC/OPSCC	235 pts; ISH for HPV and EBV; IHC biomarkers	HPV in 25% OPSCC, 11% OSCC; co-infection linked to worse survival	HPV co-infection defines adverse subgroup
Satgunaseelan et al., 2021 (12)	Australia	Comparative genomic/transcriptomic study	Identify molecular alterations specific to young (<50 years) OSCC patients	Cohort: 26 patients <50 years (17 from Sydney, 9 from TCGA) vs. 11 patients ≥50 years (TCGA)	Young OSCC patients had lower mutation burden than older ones EGFR-amplified cell lines showed active signaling and strong sensitivity to EGFR inhibitors. Genomic differences were not fully explained by smoking	EGFR amplification is a key, actionable biomarker in young OSCC. Routine testing could enable personalized therapy. EGFR inhibitors, especially afatinib, may improve outcomes in this group
Petrović et al., 2023 (160)	Serbia	Cross-sectional pilot	Prevalence HR-HPV in OSCC/OPMD/controls	90 subjects; oral swabs; real-time PCR	HPV in 16.7% OSCC, 6.7% OPMD, 0% controls; non-16/18 types	HPV may play role in OSCC; broader genotype spectrum
Anwar et al., 2024 (29)	Pakistan	Cross-sectional	HR-HPV prevalence and link with p16, habits	186 OSCC biopsies; PCR for HPV16/18; IHC p16	HPV in 3.8% OSCC; weak correlation with p16; no link with chewing habits	HR-HPV marginal in OSCC; tobacco main driver
Tangthongkum et al., 2024 (161)	Thailand	Retrospective cohort	Impact of HPV on OSCC survival	454 OSCC; multiplex PCR; survival analysis	HPV+ in 10.2%; no survival advantage; stage and treatment were main prognostic factors	HPV status not prognostic in OSCC
Becker et al., 2024 (162)	Thailand	Retrospective cohort	HPV infection and OSCC prognosis	454 OSCC; PCR and survival analysis	HPV+ in 10.2%; no survival difference; outcomes linked to stage, ECOG, treatment	HPV not prognostic in OSCC; other risk factors dominant

### 3.2 Quality and risk of bias assessment for the included articles

The methodological quality and risk of bias of the fourteen included studies were assessed using the ROBINS I Tool for observational studies (Table 3). Each study was independently evaluated by four reviewers (L.C., I.T., L.F.,M.F., and F.I.). The ROBINS I risk of bias tool included seven dominies. Disagreements between reviewers were resolved through discussion and consensus, with the involvement of additional researchers (G.D., F.I., G.M., G.F., L.L., A.D.I., and A.M.I.) as needed. A summary of the item-by-item assessment for each study is provided in Table 2. The methodological quality and potential risk of bias of the studies included in this review were carefully evaluated using the ROBINS-I tool, which is specifically designed for non-randomized observational studies. This tool examines seven key domains, including participant selection, classification of interventions, deviations from intended interventions, handling of missing data, and outcome measurement. Each study was independently assessed by a panel of four reviewers to ensure objectivity and consistency in the evaluation process. Whenever disagreements arose, they were discussed and resolved by consensus, with additional researchers contributing when necessary. This collaborative approach strengthened the reliability of the assessments.

**TABLE 3:** A tabular summary of the risk of bias assessment for 14 studies, evaluated across the seven domains of ROBINS I.

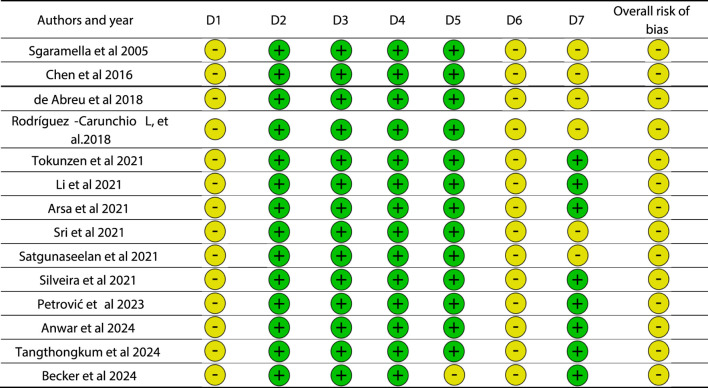

Domains	Judgement	
D1: Bias arising from the randomization process	​	​
D2: Selection of participants	High	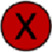
D3: Classification of interventions	Some concerns	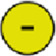
D4: Deviations from intended interventions	Low	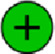
D5: Missing data	​	​
D6: Measurement of outcomes	​	​
D7: Measurement of outcomes	​	​

Overall, the risk of bias varied across studies, reflecting differences in study design, reporting quality, and methodological rigor. While some studies demonstrated low risk in several domains, others raised concerns, particularly in participant selection and intervention classification. The detailed results of this assessment are presented in tabular form (Table 3), providing a transparent overview of the strengths and limitations of each included study. This evaluation is essential for interpreting the findings of the systematic review, as it highlights both the robustness and the potential weaknesses of the available evidence.

## 4 Cases series

These two clinical cases, treated at the Department of Interdisciplinary Medicine, University of Bari ‘Aldo Moro,’ exemplify the heterogeneity of OSCC in terms of etiology, presentation, and biological behavior. The first case highlights the importance of early diagnosis and the effectiveness of minimally invasive diode laser excision in achieving complete disease control with excellent functional and aesthetic outcomes. Conversely, the second case underscores the emerging role of HPV infection in specific histological variants such as verrucous carcinoma, suggesting that viral status may influence both pathogenesis and prognosis. Taken together, these cases emphasize the need for a multidisciplinary diagnostic approach that combines clinical evaluation, imaging, histopathology, and, when appropriate, molecular testing. Such integration is essential not only to optimize patient outcomes but also to deepen our understanding of the different pathways driving oral carcinogenesis. In both presented cases, HPV16 positivity and p16 overexpression were associated with well-differentiated morphology and a non-keratinizing pattern, consistent with the biological behavior described for HPV-related squamous cell carcinomas. However, given the limited number of cases, no definitive conclusion can be drawn regarding the impact of HPV16 infection on the clinical and pathological features of oral cancer.

### 4.1 Case report 1

**FIGURE 3:**
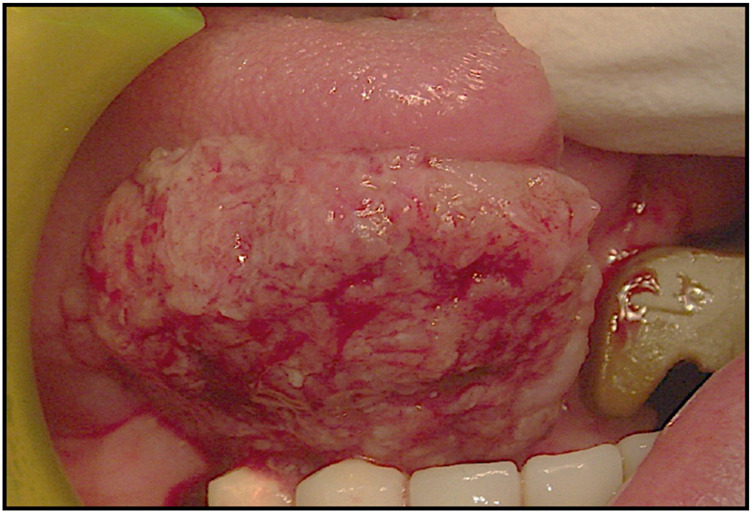
Clinical appearance of a nodular ulcerated lesion localized to the right tongue margin, also with pain and bleeding.

**FIGURE 4:**
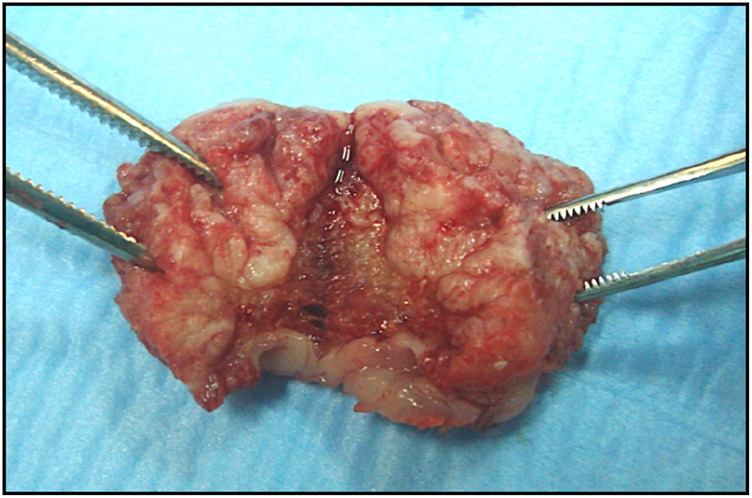
Macroscopy of the excised specimen, performed with a Diode Laser set at 3, 5–4 W in continuous wave mode.

**FIGURE 5:**
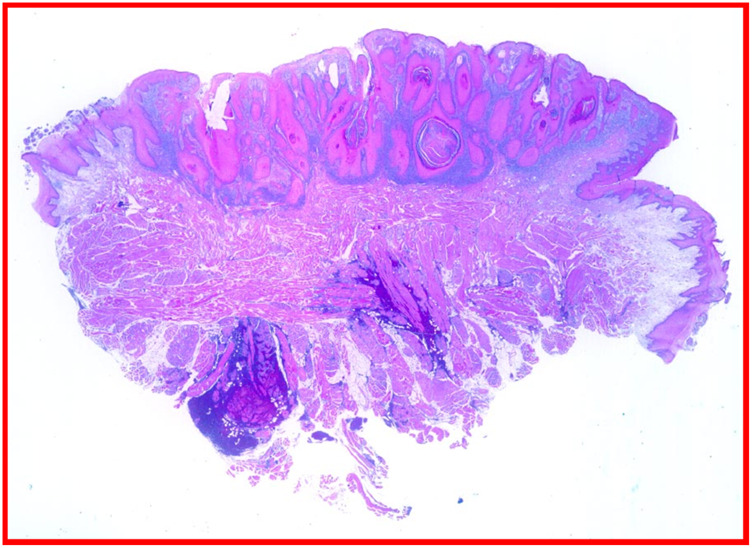
Histopathological analysis of the excised lesion confirming the diagnosis of squamous cell carcinoma (H&E).

**FIGURE 6:**
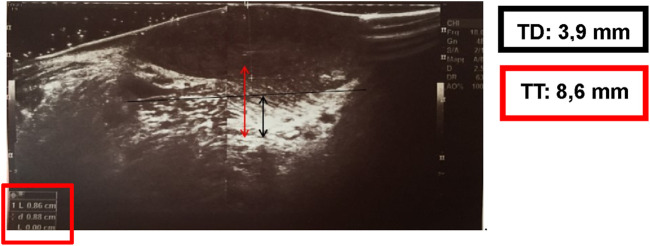
Intraoral High Definition Ultrasonography examination of the lesion appeared as a well-defined hypoechoic area measuring 8, 6 mm in Tumor Thickness and 3, 9 mm in Tumor Depth.

**FIGURE 7:**
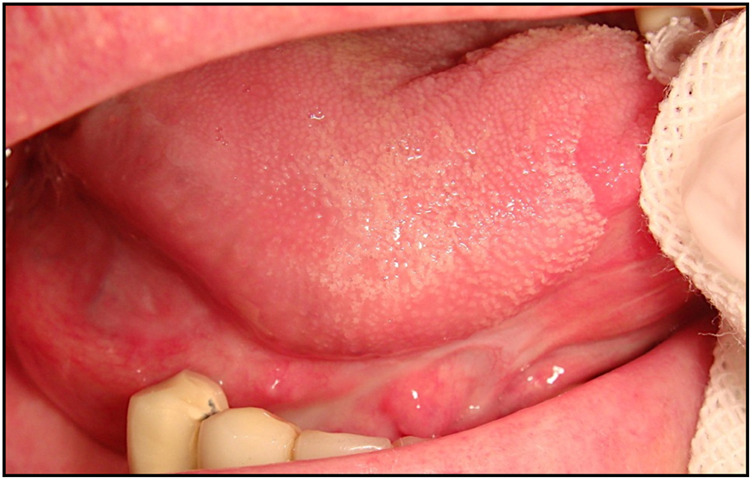
One year follow up showed complete healing of the site and absence of recurrences.

A 69-year-old male, with no history of tobacco or alcohol consumption, presented with a nodular ulcerated lesion on the right margin of the tongue, accompanied by pain and occasional bleeding. High-definition intraoral ultrasonography revealed a well-defined hypoechoic lesion measuring 8.6 mm in tumor thickness and 3.9 mm in depth. The patient underwent surgical therapy for early OC through 3D laser-guided excision. After topical staining with Lugol’s iodine and Toluidine Blue to better delineate the extension, the lesion was excised using a diode laser set at 3.5–4 W in continuous wave mode, followed by primary closure with sutures. Histopathological analysis confirmed the diagnosis of squamous cell carcinoma with keratin islands, an p16 immunohistochemistry performed to detect HPV resulted positive, also with positivity for genotype 16 HPV DNA. The postoperative course was uneventful, with complete healing observed. At 12-month follow-up, there was no evidence of recurrence or residual disease, highlighting the effectiveness of laser-guided excision in early OC (Figures 3–7).

### 4.2 Case report 2

**FIGURE 8:**
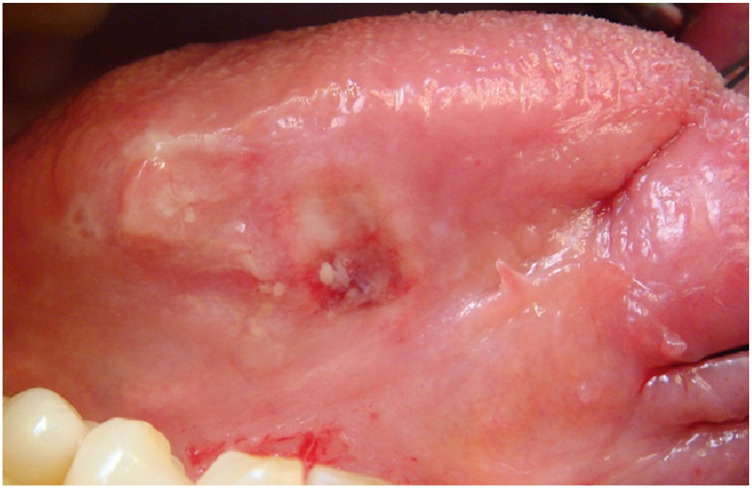
Clinical evidence of an ulcerated lesion of the right tongue margin persistent for over months.

**FIGURE 9:**
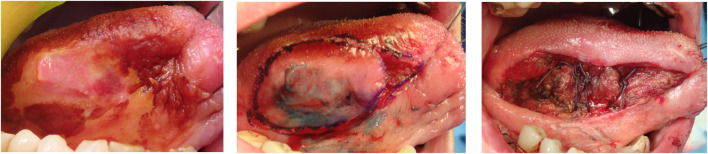
Direct application of Lugol and Toluidine Blue solution better evidenced the clinical extension of the lesion which was surgically excised with a Diode Laser (3, 5–4 W in continuous wave modality) and closure obtained with stitches.

**FIGURE 10:**
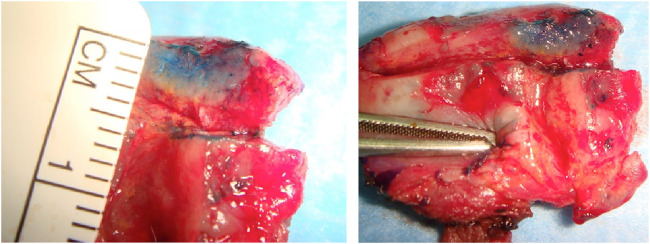
Macroscopy detail of the excised lesion which was sent for the histopathological analysis.

**FIGURE 11:**
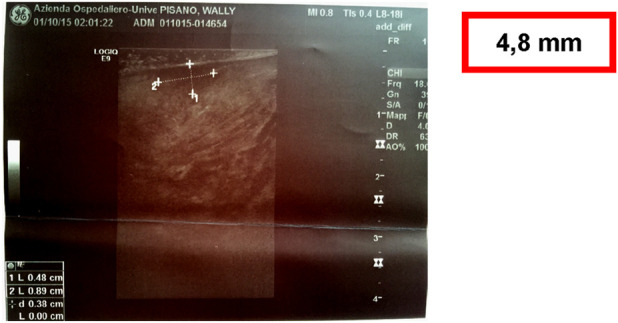
Intraoral high definition ultrasonography examination of the lesion showed an hypoechoic lesion measuring 4, 8 mm × 8.9 mm.

**FIGURE 12:**
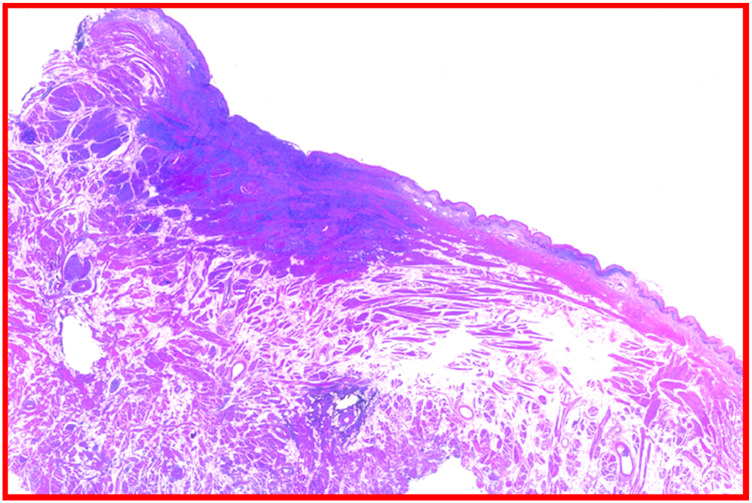
Histopathological analysis showed squamous cells patterns also with keratin islands consistent with diagnosis squamous cell carcinoma (H&E).

**FIGURE 13:**
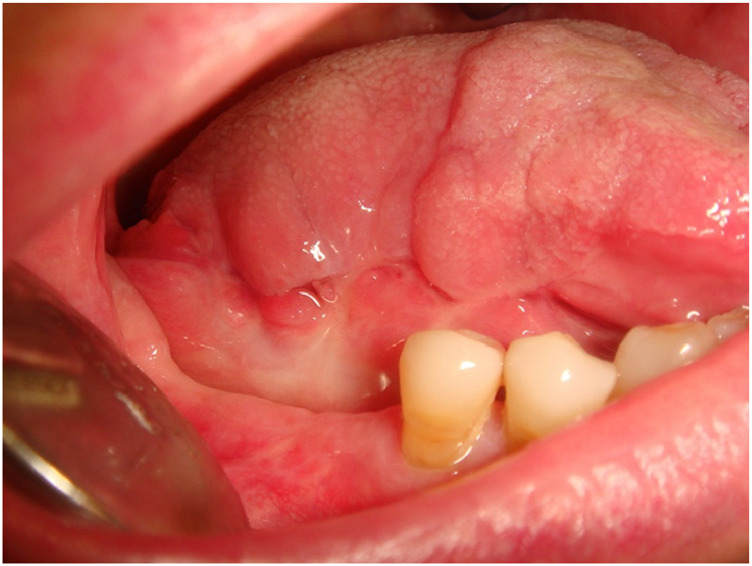
Three months follow up showed no recurrences and healing of the treated site.

A 54-year-old female, non-smoker and non-drinker, presented with a slowly enlarging verrucous lesion of the buccal mucosa, characterized clinically by thick hyperkeratosis and a warty surface. The lesion had been present for more than 6 months and was refractory to conservative management. Histopathological examination revealed a well-differentiated squamous epithelium with minimal cytological atypia, elongated rete ridges, and keratin plugs, consistent with verrucous carcinoma. HPV DNA testing was positive for genotype 16; p16 immunohistochemistry testing was positive for HPV, confirming viral involvement. The patient underwent surgical therapy for early OC through 3D laser-guided excision. The lesion was surgically excised with safety margins, and the postoperative course was uneventful. At 6-month follow-up, the patient showed no evidence of recurrence. This case illustrates the potential oncogenic role of HPV in non-conventional variants of oral squamous carcinoma and supports the importance of molecular testing in selected clinical presentations (Figures 8–13).

## 5 Discussion

### 5.1 Evidence from included studies

This systematic review integrates the findings of fifteen studies that investigated the role of HPV infection, p16 expression, and molecular alterations in OSCC across different geographic and clinical contexts. Together, these works provide a complex and sometimes contradictory picture, underscoring that the etiological and prognostic relevance of HPV in oral cavity cancer remains unsettled. Unlike in oropharyngeal squamous cell carcinoma, where HPV-driven oncogenesis is well established, the available evidence suggests that HPV plays at most a limited or context-dependent role in OSCC. A considerable group of studies strongly argued against a major contribution of HPV to oral carcinogenesis. Sgaramella et al. (2015), working in Italy, analyzed 109 cases of tongue squamous cell carcinoma and found no transcriptionally active HPV infection (152). Instead, they observed that p16 negativity was common in younger patients and associated with poorer prognosis, suggesting that p16 has prognostic significance independent of viral infection. Similarly, Chen et al. (2016) in China, examining 99 patients with OSCC and oral potentially malignant disorders, showed that many apparent HPV16/18 positive cases were in fact false positives when confirmatory sequencing was applied, reinforcing the rarity of biologically relevant HPV infection in this population (153). Marinho de Abreu et al. (2018) extended this conclusion to a Brazilian cohort, detecting HPV16 DNA in only 3.3% of OSCC samples and noting that positivity rates were similar to background prevalence in healthy individuals (154). In Japan, Tokuzen et al. (2021) identified transcriptionally active HPV16 in just one out of 100 OSCC cases and cautioned against the overuse of p16 immunohistochemistry, citing its poor specificity in this setting (156). More recently, Anwar et al. (2024) in Pakistan confirmed that HPV16/18 were present in only 3.8% of cases and highlighted the discordance between HPV DNA and p16 expression, with the latter frequently positive even in HPV-negative tumors (29). Petrović et al. (2023) in Serbia contributed a further nuance by reporting HPV DNA in 16.7% of cases, but involving non-classical high-risk genotypes (HPV33, HPV39, HPV51, HPV52, HPV59) rather than HPV16/18, suggesting that the oncogenic spectrum of HPV in OSCC, if relevant, may differ from that seen in oropharyngeal cancers. Finally, large cohort studies in Thailand by Tangthongkum et al. (2024) and Becker (2024) found HPV positivity in around 10% of OSCC but observed no prognostic correlation, reinforcing the conclusion that HPV has limited clinical significance in oral cavity cancers. In contrast, a smaller group of investigations suggested that HPV might play a more prominent role in a subset of OSCC cases (161,162). Sathya Sri et al. (2021) in India reported HPV16 positivity in as many as 35% of tumors, particularly in verrucous and well-differentiated carcinomas, arguing for a possible oncogenic role of HPV in specific histological subtypes (158). Silveira et al. (2021/2022) in Brazil found high-risk HPV in 11% of OSCC samples and noted that co-infections correlated with worse prognosis, raising the possibility that HPV could act as a modifier of tumor biology rather than as a primary etiological factor (159). Arsa et al. (2021) in Thailand added further complexity, showing that while HPV DNA was rare (4%), p16 expression correlated with better survival outcomes even outside the oropharynx. This implies that p16, despite its unreliability as a surrogate for HPV infection, may still capture biologically meaningful features of OSCC. Other studies expanded the discussion beyond HPV, emphasizing alternative prognostic and molecular biomarkers (30). Li et al. (2021), in a large Chinese cohort of 674 surgically treated OSCC patients, showed that the number of metastatic lymph nodes was the strongest independent predictor of survival, overshadowing p16 status (157). Finally, Satgunaseelan et al. (2021) highlighted the importance of non-viral molecular drivers by identifying EGFR amplification in young OSCC patients, a feature that conferred marked sensitivity to EGFR inhibitors such as afatinib (12). This study opened the way to considering precision oncology approaches targeting actionable genomic alterations rather than focusing solely on viral status.

Taken together, the fifteen studies provide a heterogeneous but informative picture. On one hand, the weight of evidence from multiple regions, including Italy, China, Brazil, Japan, Pakistan, Serbia, and Thailand, suggests that high-risk HPV16/18 are rarely detected in OSCC and often lack transcriptional activity. On the other hand, a subset of studies, particularly from India and Brazil, indicate that HPV16 may contribute to a proportion of OSCC cases, especially in specific histological contexts. Moreover, the inconsistent prognostic impact of p16 and the emergence of alternative molecular drivers such as EGFR amplification underline that OSCC should not be reduced to a binary classification of HPV-positive versus HPV-negative but instead understood within a multifactorial etiological framework.

### 5.2 Sources of heterogeneity, strengths and limitations

The heterogeneity across the fifteen studies explains much of the conflicting evidence on HPV in OSCC. Methodological variability is central: Chen et al. (2016) and Tokuzen et al. (2021) demonstrated how false positives arise without confirmatory assays, while other works (e.g., Sathya Sri 2021, Petrović 2023) used DNA detection without RNA validation, leaving uncertainty about transcriptional activity. Several groups, including Silveira (2021) and Arsa (2021), combined HPV typing with p16 immunohistochemistry but reported variable concordance (30, 159).

Geographical differences further shaped findings. While studies in China, Japan, Pakistan, and Thailand generally reported very low HPV prevalence, India (Sathya Sri 2021) and Brazil (Silveira 2021) showed higher detection rates in certain histological subtypes. Serbia (Petrović 2023) uniquely highlighted non-16/18 genotypes. These variations suggest the influence of regional viral ecology, cultural practices, and behavioral exposures (158–160). Indeed, Nopmaneepaisarn (2019), Tangthongkum (2024) and Anwar (2024) all emphasized the dominant role of betel quid, smokeless tobacco, and gutka in OSCC burden (29, 155, 161).

Strengths across the studies include large cohorts (Li 2021, Becker 2024, Tangthongkum 2024), and innovative molecular analyses (Satgunaseelan 2021). Limitations, however, are frequent: small sample sizes (Sathya Sri 2021), retrospective single-center designs, lack of functional assays, and over-reliance on p16 IHC without molecular confirmation (12, 161, 162). Importantly, no included study directly evaluated the effect of HPV vaccination on OSCC incidence, leaving a key gap.

### 5.3 Clinical and research implications

Collectively, the evidence suggests that HPV is not a major etiological driver of OSCC, unlike in oropharyngeal squamous cell carcinoma. Studies from Italy (Sgaramella 2015), China (Chen 2016), Brazil (Marinho de Abreu 2018), Japan (Tokuzen 2021), and Pakistan (Anwar 2024) all converge on very low HPV16/18 prevalence, supporting the conclusion that routine HPV or p16 testing in OSCC has limited diagnostic or prognostic utility. While p16 may carry prognostic value in some contexts (Arsa 2021), survival is more strongly determined by nodal burden (Li 2021).

Regional and behavioral contexts are critical: betel quid and tobacco use dominate in South and Southeast Asia (Nopmaneepaisarn 2019; Tangthongkum 2024; Anwar 2024), while HPV prevalence is somewhat higher in Indian and Brazilian subsets (Sathya Sri 2021; Silveira 2021). Petrović (2023) highlighted the possible oncogenic role of non-16/18 genotypes, raising questions for future surveillance.

Finally, new molecular insights highlight alternative targets for precision oncology. Satgunaseelan et al. (2021) demonstrated EGFR amplification as a therapeutically actionable driver in young patients, and Silveira et al. (2021) linked HPV co-infection to worse outcomes. These findings underscore that OSCC is primarily driven by traditional carcinogens and molecular alterations, not by HPV. Future research should prioritize standardized HPV testing, prospective multicenter cohorts, and exploration of non-viral molecular drivers, while clinical practice should focus on prevention of established risk factors and integration of actionable biomarkers.

## 6 Conclusion

Unlike oropharyngeal squamous cell carcinoma, HPV has a limited and inconsistent etiological involvement in OSCC and its verrucous variation. The high-risk HPV genotypes, specifically HPV16 and HPV18, are quite uncommon, and transcriptionally active infections are still in a somewhat unique form. This suggests that HPV is not the only contributing factor in the development of OC, but rather one among multiple etiological determinants. The primary risk factors are traditional ones, such as alcohol and tobacco use, as well as local customs like gutka and betel quid. However, information from particular geographical areas (like Brazil and India) and histological subtypes (such VC and well-differentiated OSCC) indicates that HPV might be involved in a portion of cases. The interpretation is further complicated by the potential participation of non-16/18 genotypes. Crucially, prognostic analyses consistently show that molecular changes, including EGFR amplification, clinical stage, and nodal burden are considerably more important for patient outcomes than p16 or HPV status. Even though p16 immunohistochemistry can sometimes predict outcomes, it is not a trustworthy indicator of HPV infection. All things considered, OSCC is a complex illness in which HPV plays, at most, a supporting or modifying function.

## 7 Future perspective and limitations

In order to validate transcriptionally active infections and prevent false positives, future research should place a high priority on methodological uniformity and use assays based on both DNA and RNA. To ascertain whether there is a unique subset of HPV-related OSCC and to ascertain its clinical and biological significance, prospective multicenter cohorts are required.

It is necessary to investigate regional variations in HPV prevalence and genotype distribution, especially in relation to non-16/18 high-risk forms. Clinically speaking, combining viral status with non-viral molecular indicators like co-infection patterns or EGFR amplification may improve prognosis models and direct precision treatment. Long-term epidemiological studies comparing vaccinated and unvaccinated cohorts are necessary to evaluate the possible effect of HPV vaccination on OSCC incidence at the population level, as this effect is still unknown. Lastly, in order to create more efficient, customized treatment plans, therapeutic research should go beyond HPV and concentrate on modifiable genetic changes.

## Data Availability

The original contributions presented in the study are included in the article/supplementary material, further inquiries can be directed to the corresponding author.

## Appendix A

Pub Med Query:

(“oral carcinoma” [Title/Abstract] OR “oral squamous cell carcinoma” [Title/Abstract] OR “oral verrucous carcinoma” [Title/Abstract]) AND (“human papillomavirus” [Title/Abstract] OR HPV [Title/Abstract])

Scopus query:

(“oral carcinoma” [Title/Abstract] OR “oral squamous cell carcino-ma” [Title/Abstract] OR “oral verrucous carcinoma” [Title/Abstract]) AND (“human papillomavirus” [Title/Abstract] OR HPV [Title/Abstract]);

Web of Science query:

TS = (“oral carcinoma” OR “oral squamous cell carcinoma” OR “oral verrucous carcinoma”) AND TS = (“human papillomavirus” OR HPV).

## References

[1] Science Direct. Oral cancer: recent breakthroughs in pathology and therapeutic approaches - ScienceDirect (2025). Available online at: https://www.sciencedirect.com/science/article/pii/S2772906024005247 (Accessed on September 22, 2025).

[2] Tan Y Wang Z Xu M Li B Huang Z Qin S Oral squamous cell carcinomas: state of the field and emerging directions. Int J Oral Sci (2023) 15:44. 10.1038/s41368-023-00249-w 37736748 PMC10517027

[3] Al-Jamaei AAH Helder MN Forouzanfar T Brakenhoff RH Leemans CR de Visscher JGAM Age-group-specific trend analyses of oropharyngeal squamous cell carcinoma incidence from 1989 to 2018 and risk factors profile by age-group in 2015-2018: a population-based study in the Netherlands. Eur J Cancer Prev (2022) 31:158–65. 10.1097/CEJ.0000000000000678 34267108

[4] McAllister P Affleck A Manickavasagam J Evans A . Aggressive cutaneous squamous cell carcinoma arising from a human papillomavirus-infected epidermoid cyst of the conchal bowl. Clin Exp Dermatol (2018) 43:201–3. 10.1111/ced.13305 29226981

[5] Fulcher CD Haigentz MJ Ow TJ Education Committee of the American Head and Neck Society AHNS. AHNS series: do you know your guidelines? Principles of treatment for locally advanced or unresectable head and neck squamous cell carcinoma. Head Neck (2018) 40:676–86. 10.1002/hed.25025 29171929 PMC5849482

[6] Eickelschulte S Starus A Murray DH Keyser KA Schauer O Guellert S Analytical and clinical performance validation of HPV-SEQ, a novel NGS-based liquid biopsy platform for detection and quantification of human papilloma virus circulating tumor DNA. Oral Oncol (2025) 167:107445. 10.1016/j.oraloncology.2025.107445 40578243 PMC13729803

[7] Mazurek AM Rutkowski T Fiszer-Kierzkowska A Małusecka E Składowski K . Assessment of the total cfDNA and HPV16/18 detection in plasma samples of head and neck squamous cell carcinoma patients. Oral Oncol (2016) 54:36–41. 10.1016/j.oraloncology.2015.12.002 26786940

[8] Khowal S Naqvi SH Monga S Jain SK Wajid S . Assessment of cellular and serum proteome from tongue squamous cell carcinoma patient lacking addictive proclivities for tobacco, betel nut, and alcohol: case study. J Cell Biochem (2018) 119:5186–221. 10.1002/jcb.26554 29236289

[9] Daskalopoulos AG Avgoustidis D Chaisuparat R Karanikou M Lazaris AC Sklavounou A Assessment of TLR4 and TLR9 signaling and correlation with human papillomavirus status and histopathologic parameters in oral tongue squamous cell carcinoma. Oral Surg Oral Med Oral Pathol Oral Radiol (2020) 129:493–513. 10.1016/j.oooo.2020.01.001 32173390

[10] Pandey P Ralli M Dixit A Agarwal S Chaturvedi V Sawhney A Assessment of immunohistochemical expression of P16 in head and neck squamous cell carcinoma and their correlation with clinicopathological parameters. J Oral Maxillofac Pathol (2021) 25:74–81. 10.4103/jomfp.JOMFP_252_20 34349415 PMC8272494

[11] Gelwan E Malm I-J Khararjian A Fakhry C Bishop JA Westra WH . Nonuniform distribution of high-risk human papillomavirus in squamous cell carcinomas of the oropharynx: rethinking the anatomic boundaries of oral and oropharyngeal carcinoma from an oncologic HPV perspective. Am J Surg Pathol (2017) 41:1722–8. 10.1097/PAS.0000000000000929 28877058

[12] Satgunaseelan L Porazinski S Strbenac D Istadi A Willet C Chew T Oral squamous cell carcinoma in young patients show higher rates of EGFR amplification: implications for novel personalized therapy. Front Oncol (2021) 11:750852. 10.3389/fonc.2021.750852 34912708 PMC8666981

[13] Vigneswaran N Williams MD . Epidemiologic trends in head and neck cancer and aids in diagnosis. Oral Maxillofac Surg Clin North Am (2014) 26:123–41. 10.1016/j.coms.2014.01.001 24794262 PMC4040236

[14] Salazar C Calvopiña D Punyadeera C . miRNAs in human papilloma virus associated oral and oropharyngeal squamous cell carcinomas. Expert Rev Mol Diagn (2014) 14:1033–40. 10.1586/14737159.2014.960519 25222489

[15] Poh CF Zhang L Lam WL Zhang X An D Chau C A high frequency of allelic loss in oral verrucous lesions may explain malignant risk. Lab Invest (2001) 81:629–34. 10.1038/labinvest.3780271 11304582

[16] Chitsike L Yuan C-H Roy A Boyle K Duerksen-Hughes PJ . A high-content AlphaScreen^TM^ identifies E6-Specific small molecule inhibitors as potential therapeutics for HPV(+) head and neck squamous cell carcinomas. Oncotarget (2021) 12:549–61. 10.18632/oncotarget.27908 33796223 PMC7984827

[17] Zhang Q Chen Y Hu S-Q Pu Y-M Zhang K Wang Y-X . A HPV16-Related prognostic indicator for head and neck squamous cell carcinoma. Ann Transl Med (2020) 8:1492. 10.21037/atm-20-6338 33313237 PMC7729314

[18] Jayasooriya PR Tilakaratne WM Mendis BRRN Lombardi T . A literature review on oral basaloid squamous cell carcinomas, with special emphasis on etiology. Ann Diagn Pathol (2013) 17:547–51. 10.1016/j.anndiagpath.2013.09.001 24157420

[19] Almadori G Cadoni G Cattani P Posteraro P Scarano E Ottaviani F Detection of human papillomavirus DNA in laryngeal squamous cell carcinoma by polymerase chain reaction. Eur J Cancer (1996) 32A:783–8. 10.1016/0959-8049(95)00628-1 9081354

[20] O’Neill SH Newkirk KM Anis EA Brahmbhatt R Frank LA Kania SA . Detection of human papillomavirus DNA in feline premalignant and invasive squamous cell carcinoma. Vet Dermatol (2011) 22:68–74. 10.1111/j.1365-3164.2010.00912.x 20609206

[21] Maitland NJ Cox MF Lynas C Prime SS Meanwell CA Scully C . Detection of human papillomavirus DNA in biopsies of human oral tissue. Br J Cancer (1987) 56:245–50. 10.1038/bjc.1987.185 2822070 PMC2002211

[22] Arndt O Brock J Kundt G Müllender A . Detection of human papillomavirus DNA in formalin fixed invasive squamous cell carcinoma of the larynx with polymerase chain reaction (PCR). Laryngorhinootologie (1994) 73:527–32. 10.1055/s-2007-997187 7802882

[23] Gargiulo Isacco C Balzanelli MG Garzone S Lorusso M Inchingolo F Nguyen KCD Alterations of vaginal microbiota and Chlamydia trachomatis as crucial Co-Causative factors in cervical cancer genesis procured by HPV. Microorganisms (2023) 11:662. 10.3390/microorganisms11030662 36985236 PMC10053692

[24] Inchingolo F Martelli FS Gargiulo Isacco C Borsani E Cantore S Corcioli F Chronic periodontitis and immunity, towards the implementation of a personalized medicine: a translational research on gene single nucleotide polymorphisms (SNPs) linked to chronic oral dysbiosis in 96 caucasian patients. Biomedicines (2020) 8:115. 10.3390/biomedicines8050115 32397555 PMC7277173

[25] Bordea IR Xhajanka E Candrea S Bran S Onişor F Inchingolo AD Coronavirus (SARS-CoV-2) pandemic: future challenges for dental practitioners. Microorganisms (2020) 8:1704. 10.3390/microorganisms8111704 33142764 PMC7694165

[26] Aldelaimi TN Khalil AA . Diagnosis and surgical management of nasopalatine duct cysts. J Craniofac Surg (2012) 23:e472–474. 10.1097/SCS.0b013e318258764b 22976713

[27] Amam MA Abdo A Alnour A Amam A Jaafo MH . Comparison of calcium sulfate and tricalcium phosphate in bone grafting after sinus lifting for dental implantation: a randomized controlled trial. Dent Med Probl (2023) 60:239–46. 10.17219/dmp/151983 37350471

[28] Amritzer MA Muntlin Å Berg LM Göransson KE . Nursing staff ratio and skill mix in Swedish emergency departments: a national cross-sectional benchmark study. J Nurs Manag (2021) 29:2594–602. 10.1111/jonm.13424 34273138

[29] Anwar N Chundriger Q Awan S Moatter T Ali TS Abdul Rasheed M Prevalence of high-risk human papillomavirus in oral squamous cell carcinoma with or without chewing habits. PLoS One (2024) 19:e0300354. 10.1371/journal.pone.0300354 38691559 PMC11062528

[30] Arsa L Siripoon T Trachu N Foyhirun S Pangpunyakulchai D Sanpapant S Discrepancy in P16 expression in patients with HPV-associated head and neck squamous cell carcinoma in Thailand: clinical characteristics and survival outcomes. BMC Cancer (2021) 21:504. 10.1186/s12885-021-08213-9 33957888 PMC8101232

[31] Bae S-H Fabry D . Assessing the relationships between nurse work hours/overtime and nurse and patient outcomes: systematic literature review. Nurs Outlook (2014) 62:138–56. 10.1016/j.outlook.2013.10.009 24345613

[32] Bajaj V Kolte AP Kolte R Bawankar PV . Comparative evaluation of immediate implant placement and provisionalization (IIPP) with and without a concentrated growth factor-enriched bone graft: a randomized controlled trial. Dent Med Probl (2025) 62:449–59. 10.17219/dmp/170045 40626839

[33] Mishra MK Gupta S Sehgal S . Assessing long non-coding RNAs in tobacco-associated oral cancer. Curr Cancer Drug Targets (2022) 22:879–88. 10.2174/1568009622666220623115234 35747968

[34] Bartemes KR Gochanour BR Routman DM Ma DJ Doering KA Burger KN Assessing the capacity of methylated DNA markers of cervical squamous cell carcinoma to discriminate oropharyngeal squamous cell carcinoma in human papillomavirus mediated disease. Oral Oncol (2023) 146:106568. 10.1016/j.oraloncology.2023.106568 37717549 PMC10591712

[35] Alsharif MT Alsahafi E . Assessing the knowledge of HPV-associated oropharyngeal squamous cell carcinoma, HPV vaccination, and practice scope among Saudi dental students in the Western region. Healthcare (Basel) (2024) 12:905. 10.3390/healthcare12090905 38727462 PMC11083101

[36] More P Kheur S Patekar D Kheur M Gupta AA Raj AT Assessing the nature of the association of human papillomavirus in oral cancer with and without known risk factors. Transl Cancer Res (2020) 9:3119–25. 10.21037/tcr.2020.03.81 35117675 PMC8798937

[37] Ralli M Singh S Yadav SPS Sharma N Verma R Sen R . Assessment and clinicopathological correlation of P16 expression in head and neck squamous cell carcinoma. J Cancer Res Ther (2016) 12:232–7. 10.4103/0973-1482.151447 27072243

[38] Shankar K Walker SE . Analysis of divergent gene expression between HPV + and HPV- head and neck squamous cell carcinoma patients. Infect Agent Cancer (2025) 20:31. 10.1186/s13027-025-00663-1 40400005 PMC12096591

[39] Flach S Kumbrink J Walz C Hess J Drexler G Belka C Analysis of genetic variants of frequently mutated genes in human papillomavirus-negative primary head and neck squamous cell carcinoma, resection margins, local recurrences and corresponding circulating cell-free DNA. J Oral Pathol Med (2022) 51:738–46. 10.1111/jop.13338 35895622

[40] Li C-D Zhang W-Y Wu M-H Zhang S-W Zhou B-L Zhu L Analysis of high risk factors associated with cervical intraepithelial neoplasia in married women aged 25 - 54 years in beijing between 2007 - 2008. Zhonghua Fu Chan Ke Za Zhi (2010) 45:757–61. 21176557

[41] Woods KV Shillitoe EJ Spitz MR Schantz SP Adler-Storthz K . Analysis of human papillomavirus DNA in oral squamous cell carcinomas. J Oral Pathol Med (1993) 22:101–8. 10.1111/j.1600-0714.1993.tb01038.x 8387592

[42] Cortezzi SS Provazzi PJ Sobrinho JS Mann-Prado JC Reis PMP de Freitas SEN Analysis of human papillomavirus prevalence and TP53 polymorphism in head and neck squamous cell carcinomas. Cancer Genet Cytogenet (2004) 150:44–9. 10.1016/j.cancergencyto.2003.07.010 15041222

[43] Xu S-M Shi C-J Xia R-H Wang L-Z Tian Z Ye W-M Analysis of immunological characteristics and genomic alterations in HPV-positive oropharyngeal squamous cell carcinoma based on PD-L1 expression. Front Immunol (2021) 12:798424. 10.3389/fimmu.2021.798424 35145511 PMC8821172

[44] Zhang Y Qiu K Ren J Zhao Y Cheng P . Roles of human papillomavirus in cancers: oncogenic mechanisms and clinical use. Signal Transduct Target Ther (2025) 10 (1):44. 10.1038/s41392-024-02083-w 39856040 PMC11760352

[45] Pavelescu LA Mititelu-Zafiu NL Mindru DE Vladareanu R Curici A . Molecular insights into HPV-driven cervical cancer: oncoproteins, immune evasion, and epigenetic modifications. Microorganisms (2025) 13:1000. 10.3390/microorganisms13051000 40431173 PMC12113743

[46] Asiaf A Ahmad ST Mohammad SO Zargar MA . Review of the current knowledge on the epidemiology, pathogenesis, and prevention of human papillomavirus infection. Eur J Cancer Prev (2014) 23:206–24. 10.1097/CEJ.0b013e328364f273 24129107

[47] Mittal S Banks L . Molecular mechanisms underlying human papillomavirus E6 and E7 oncoprotein-induced cell transformation. Mutat Research/Reviews Mutat Res (2017) 772:23–35. 10.1016/j.mrrev.2016.08.001 28528687

[48] Prigge ES von Knebel Doeberitz M Reuschenbach M . Clinical relevance and implications of HPV-induced neoplasia in different anatomical locations. Mutat Research/Reviews Mutat Res (2017) 772:51–66. 10.1016/j.mrrev.2016.06.005 28528690

[49] MDPI. From viral infection to genome reshaping: the triggering role of HPV integration in cervical cancer (2025). Available online at: https://www.mdpi.com/1422-0067/26/18/9214 (Accessed on September 22, 2025). 10.3390/ijms26189214 PMC12470990 41009776

[50] Baba SK Alblooshi SSE Yaqoob R Behl S Al Saleem M Rakha EA Human papilloma virus (HPV) mediated cancers: an insightful update. J Transl Med (2025) 23 (1):483. 10.1186/s12967-025-06470-x 40301924 PMC12039116

[51] Nespeca G Grest P Rosenkrantz WS Ackermann M Favrot C . Detection of novel papillomaviruslike sequences in paraffin-embedded specimens of invasive and *in situ* squamous cell carcinomas from cats. Am J Vet Res (2006) 67:2036–41. 10.2460/ajvr.67.12.2036 17144807

[52] Ong JJ Read TRH Vodstrcil LA Walker S Chen M Bradshaw CS Detection of oral human papillomavirus in HIV-positive men who have sex with men 3 years after baseline: a follow up cross-sectional study. PLoS One (2014) 9:e102138. 10.1371/journal.pone.0102138 25033212 PMC4102527

[53] Munday JS Howe L French A Squires RA Sugiarto H . Detection of papillomaviral DNA sequences in a feline oral squamous cell carcinoma. Res Vet Sci (2009) 86:359–61. 10.1016/j.rvsc.2008.07.005 18715602

[54] Sichero L Gonçalves MG Bettoni F Coser EM Mota G Nunes RAL Detection of serum biomarkers of HPV-16 driven oropharynx and oral cavity cancer in Brazil. Oral Oncol (2024) 149:106676. 10.1016/j.oraloncology.2023.106676 38150987

[55] Wang Y Springer S Mulvey CL Silliman N Schaefer J Sausen M Detection of somatic mutations and HPV in the saliva and plasma of patients with head and neck squamous cell carcinomas. Sci Transl Med (2015) 7:293ra104. 10.1126/scitranslmed.aaa8507 26109104 PMC4587492

[56] Wang J Li J Huang H Fu Y . Detection of the E7 transform gene of human papilloma virus type 16 in human oral squamous cell carcinoma. Chin J Dent Res (1998) 1:35–7. 10557171

[57] Shimizu M Adachi A Zheng S Matsunaga J Kusakari Y Tagami H Detection of various types of human papillomavirus DNA, mainly belonging to the cutaneous-group, more frequently in normal tissue than in squamous cell carcinomas of the lip. J Dermatol Sci (2004) 36:33–9. 10.1016/j.jdermsci.2004.07.005 15488703

[58] Syrjänen S . Oral manifestations of human papillomavirus infections. Eur J Oral Sci (2018) 126(Suppl. 1):49–66. 10.1111/eos.12538 30178562 PMC6174935

[59] Greer ROJ . Oral manifestations of smokeless tobacco use. Otolaryngol Clin North Am (2011) 44:31–56. 10.1016/j.otc.2010.09.002 21093622

[60] Lissoni A Agliardi E Peri A Marchioni R Abati S . Oral microbiome and mucosal trauma as risk factors for oral cancer: beyond alcohol and tobacco. A literature review. J Biol Regul Homeost Agents (2020) 34:11–8. 33386052

[61] Sukmana BI Saleh RO Najim MA Al-Ghamdi HS Achmad H Al-Hamdani MM Oral microbiota and oral squamous cell carcinoma: a review of their relation and carcinogenic mechanisms. Front Oncol (2024) 14:1319777. 10.3389/fonc.2024.1319777 38375155 PMC10876296

[62] Femiano F Gombos F Scully C . Oral proliferative verrucous leukoplakia (PVL); open trial of surgery compared with combined therapy using surgery and methisoprinol in papillomavirus-related PVL. Int J Oral Maxillofac Surg (2001) 30:318–22. 10.1054/ijom.2001.0066 11518355

[63] Lutzner MA Blanchet-Bardon C . Oral retinoid treatment of human papillomavirus type 5-Induced epidermodysplasia verruciformis. N Engl J Med (1980) 302:1091. 10.1056/NEJM198005083021919 6245361

[64] Author Anonymous . Oral rinses may help detect human papillomavirus-positive head, neck cancers. J Am Dent Assoc (2008) 139:1588. 10.14219/jada.archive.2008.0096 19047664

[65] Researchgate. (PDF) Co-Factors related to the causal relationship between human papillomavirus and invasive cervical cancer in Honduras (2025). Available online at: https://www.researchgate.net/publication/12289380_Co-factors_related_to_the_causal_relationship_between_human_papillomavirus_and_invasive_cervical_cancer_in_Honduras (Accessed on September 22, 2025). 10.1093/ije/29.5.817 11034963

[66] Alhamlan FS Alfageeh MB Al Mushait MA Al-Badawi IA Al-Ahdal MN . Human papillomavirus-associated cancers. Adv Exp Med Biol (2021) 1313:1–14. 10.1007/978-3-030-67452-6_1 34661888

[67] Akagi K Li J Broutian TR Padilla-Nash H Xiao W Jiang B Genome-wide analysis of HPV integration in human cancers reveals recurrent, focal genomic instability. Genome Res (2014) 24:185–99. 10.1101/gr.164806.113 24201445 PMC3912410

[68] PubMed. Human papillomavirus integration induces oncogenic host gene fusions in oropharyngeal cancers - PubMed (2025). Available online at: https://pubmed.ncbi.nlm.nih.gov/40358390/(Accessed on September 22, 2025). 10.1158/2159-8290.CD-24-1535 PMC12315581 40358390

[69] Prins R Fernandez DJ Akbari O Da Silva DM Kast WM . HPV16 E6 and E7 expressing cancer cells suppress the antitumor immune response by upregulating KLF2-Mediated IL-23 expression in macrophages. J Immunother Cancer (2025) 13:e011915. 10.1136/jitc-2025-011915 40829900 PMC12366621

[70] PubMed. Differential tumor immune microenvironment coupled with tumor progression or tumor eradication in HPV-antigen expressing squamous cell carcinoma (SCC) models - PubMed (2025). Available online at: https://pubmed.ncbi.nlm.nih.gov/39055715/ (Accessed on September 22, 2025). 10.3389/fimmu.2024.1405318 PMC11269233 39055715

[71] Nikitina A Kiriy D Tyshevich A Tychinin D Antysheva Z Sobol A Viral transcript and tumor immune microenvironment-based transcriptomic profiling of HPV-associated head and neck squamous cell carcinoma identifies subtypes associated with prognosis. Viruses (2024) 17:4. 10.3390/v17010004 39861794 PMC11769425

[72] WHO. Publication of the WHO classification of tumours In: Head and neck tumours (2025) 5th ed., Vol. 9.

[73] Slootweg PJ El-Naggar AK . World health organization 4th edition of head and neck tumor classification: insight into the consequential modifications. Virchows Arch (2018) 472:311–3. 10.1007/s00428-018-2320-6 29450648

[74] Fakhry C Lacchetti C Rooper LM Jordan RC Rischin D Sturgis EM Human papillomavirus testing in head and neck carcinomas: ASCO clinical practice guideline endorsement of the college of American pathologists guideline. J Clin Oncol (2018) 36:3152–61. 10.1200/JCO.18.00684 30188786

[75] Kwon HJ Brasch HD Benison S Marsh RW Itinteang T Titchener GW Changing prevalence and treatment outcomes of patients with P16 human papillomavirus related oropharyngeal squamous cell carcinoma in New Zealand. Br J Oral Maxillofac Surg (2016) 54:898–903. 10.1016/j.bjoms.2016.05.033 27371339

[76] Alramadhan SA Fitzpatrick SG Bhattacharyya I Islam MN Cohen DM . Changing trends in benign human papillomavirus (HPV) related epithelial neoplasms of the oral cavity: 1995-2015. Head Neck Pathol (2022) 16:738–45. 10.1007/s12105-022-01426-9 35257322 PMC9424415

[77] Bosch FX Qiao Y-L Castellsagué X . CHAPTER 2 the epidemiology of human papillomavirus infection and its association with cervical cancer. Int J Gynaecol Obstet (2006) 94(Suppl. 1):S8–S21. 10.1016/S0020-7292(07)60004-6 29644633

[78] Gillison ML Shah KV . Chapter 9: role of mucosal human papillomavirus in nongenital cancers. J Natl Cancer Inst Monogr (2003) 31:57–65. 10.1093/oxfordjournals.jncimonographs.a003484 12807947

[79] Jiarpinitnun C Larbcharoensub N Pattaranutaporn P Chureemas T Juengsamarn J Trachu N Characteristics and impact of HPV-associated P16 expression on head and neck squamous cell carcinoma in Thai patients. Asian Pac J Cancer Prev (2020) 21:1679–87. 10.31557/APJCP.2020.21.6.1679 32592364 PMC7568885

[80] Ngamphaiboon N Chureemas T Siripoon T Arsa L Trachu N Jiarpinitnun C Characteristics and impact of programmed death-ligand 1 expression, CD8+ tumor-infiltrating lymphocytes, and P16 status in head and neck squamous cell carcinoma. Med Oncol (2019) 36:21. 10.1007/s12032-018-1241-1 30666437

[81] Ow TJ Mehta V Li D Thomas C Shrivastava N Kawachi N Characterization of a diverse set of conditionally reprogrammed head and neck cancer cell cultures. Laryngoscope (2024) 134:2748–56. 10.1002/lary.31236 38288866 PMC12007172

[82] Bellocchio L Dipalma G Inchingolo AM Inchingolo AD Ferrante L Del Vecchio G COVID-19 on oral health: a new bilateral connection for the pandemic. Biomedicines (2023) 12:60. 10.3390/biomedicines12010060 38255167 PMC10813615

[83] Inchingolo AD Malcangi G Ceci S Patano A Corriero A Vimercati L Effectiveness of SARS-CoV-2 vaccines for Short- and long-term immunity: a general overview for the pandemic contrast. Int J Mol Sci (2022) 23:8485. 10.3390/ijms23158485 35955621 PMC9369331

[84] Balzanelli MG Distratis P Lazzaro R Pham VH Del Prete R Mosca A From pathogens to cancer: are cancer cells evolved mitochondrial super cells? Diagnostics (2023) 13:813. 10.3390/diagnostics13040813 36832301 PMC9954806

[85] Topi S Santacroce L Bottalico L Ballini A Inchingolo AD Dipalma G Gastric cancer in history: a perspective interdisciplinary study. Cancers (Basel) (2020) 12:264. 10.3390/cancers12020264 31978985 PMC7072612

[86] Del Prete R Nesta D Triggiano F Lorusso M Garzone S Vitulano L Human papillomavirus carcinogenicity and the need of new perspectives: thoughts from a retrospective analysis on human papillomavirus outcomes conducted at the hospital university of Bari, apulia, Italy, between 2011 and 2022. Diagnostics (2024) 14:968. 10.3390/diagnostics14090968 38732382 PMC11083870

[87] Di Lorenzo L Inchingolo F Pipoli A Cassano F Maggiore ME Inchingolo AM Mixed-dust pneumoconiosis in a dental technician: a multidisciplinary diagnosis case report. BMC Pulm Med (2022) 22:161. 10.1186/s12890-022-01948-6 35477357 PMC9044673

[88] Gayathri PS M B Ramani P J M Jeyakumaran S Raman P . Oral squamous cell carcinoma of the right buccal mucosa: a case report. Cureus (2024) 16:e59571. 10.7759/cureus.59571 38826907 PMC11144300

[89] Katz J Islam MN Bhattacharyya I Sandow P Moreb JS . Oral squamous cell carcinoma positive for P16/Human papilloma virus in post allogeneic stem cell transplantation: 2 cases and review of the literature. Oral Surg Oral Med Oral Pathol Oral Radiol (2014) 118:e74–78. 10.1016/j.oooo.2014.05.025 25151594

[90] King M Chatelain K Farris D Jensen D Pickup J Swapp A Oral squamous cell carcinoma proliferative phenotype is modulated by proanthocyanidins: a potential prevention and treatment alternative for oral cancer. BMC Complement Altern Med (2007) 7:22. 10.1186/1472-6882-7-22 17578576 PMC1914364

[91] de Spíndula-Filho JV da Cruz AD Oton-Leite AF Batista AC Leles CR de Cássia Gonçalves Alencar R Oral squamous cell carcinoma *versus* oral verrucous carcinoma: an approach to cellular proliferation and negative relation to human papillomavirus (HPV). Tumour Biol (2011) 32:409–16. 10.1007/s13277-010-0135-4 21136231

[92] Scully C . Oral squamous cell carcinoma; from an hypothesis about a virus, to concern about possible sexual transmission. Oral Oncol (2002) 38:227–34. 10.1016/s1368-8375(01)00098-7 11978544

[93] Hoover AC Strand GL Nowicki PN Anderson ME Vermeer PD Klingelhutz AJ Impaired PTPN13 phosphatase activity in spontaneous or HPV-induced squamous cell carcinomas potentiates oncogene signaling through the MAP kinase pathway. Oncogene (2009) 28:3960–70. 10.1038/onc.2009.251 19734941 PMC2785129

[94] Subramaniam N Thankappan K Anand A Balasubramanian D Iyer S . Implementing American joint committee on cancer 8(Th) edition for head-and-neck cancer in India: context, feasibility, and practicality. Indian J Cancer (2018) 55:4–8. 10.4103/ijc.IJC_475_17 30147086

[95] Kumari S Mishra S Ali W Singh US Shabbir N Kumar V Implication of circulating miRNAs as potential diagnostic biomarker in oropharyngeal squamous cell carcinoma: association with human papilloma virus. Oral Oncol (2025) 165:107305. 10.1016/j.oraloncology.2025.107305 40286701

[96] Pham VH Pham HT Balzanelli MG Distratis P Lazzaro R Nguyen QV Multiplex RT real-time PCR based on target failure to detect and identify different variants of SARS-CoV-2: a feasible method that can be applied in clinical laboratories. Diagnostics (Basel) (2023) 13:1364. 10.3390/diagnostics13081364 37189465 PMC10137618

[97] Inchingolo F Santacroce L Ballini A Topi S Dipalma G Haxhirexha K Oral cancer: a historical review. Int J Environ Res Public Health (2020) 17:3168. 10.3390/ijerph17093168 32370133 PMC7246763

[98] Ballini A Dipalma G Isacco CG Boccellino M Di Domenico M Santacroce L Oral microbiota and immune system crosstalk: a translational research. Biology (Basel) (2020) 9:131. 10.3390/biology9060131 32560235 PMC7344575

[99] Mancini A Chirico F Inchingolo AM Piras F Colonna V Marotti P Osteonecrosis of the jaws associated with Herpes zoster infection: a systematic review and a rare case report. Microorganisms (2024) 12:1506. 10.3390/microorganisms12081506 39203349 PMC11356100

[100] Santacroce L Inchingolo F Topi S Del Prete R Di Cosola M Charitos IA Potential beneficial role of probiotics on the outcome of COVID-19 patients: an evolving perspective. Diabetes Metab Syndr (2021) 15:295–301. 10.1016/j.dsx.2020.12.040 33484986 PMC7804381

[101] Inchingolo AD Inchingolo AM Bordea IR Malcangi G Xhajanka E Scarano A SARS-CoV-2 disease through viral genomic and receptor implications: an overview of diagnostic and immunology breakthroughs. Microorganisms (2021) 9:793. 10.3390/microorganisms9040793 33920179 PMC8070527

[102] Macha MA Wani NA Ganai RA Bhat AA Hamid A Hashem S Recent advances in head and neck tumor microenvironment-based therapy. Adv Exp Med Biol (2020) 1296:11–31. 10.1007/978-3-030-59038-3_2 34185284

[103] Curado MP Hashibe M . Recent changes in the epidemiology of head and neck cancer. Curr Opin Oncol (2009) 21:194–200. 10.1097/CCO.0b013e32832a68ca 19363341

[104] Cortelazzi B Verderio P Ciniselli CM Pizzamiglio S Bossi P Gloghini A Receptor tyrosine kinase profiles and human papillomavirus status in oropharyngeal squamous cell carcinoma. J Oral Pathol Med (2015) 44:734–45. 10.1111/jop.12301 25495427

[105] Rasheed K Sveinbjørnsson B Moens U . Reciprocal transactivation of merkel cell polyomavirus and high-risk human papillomavirus promoter activities and increased expression of their oncoproteins. Virol J (2021) 18:139. 10.1186/s12985-021-01613-0 34217322 PMC8254899

[106] Kelly JR Park HS An Y Yarbrough WG Contessa JN Decker R Upfront surgery *versus* definitive chemoradiotherapy in patients with human papillomavirus-associated oropharyngeal squamous cell cancer. Oral Oncol (2018) 79:64–70. 10.1016/j.oraloncology.2018.02.017 29598952

[107] Huebbers CU Verhees F Poluschkin L Olthof NC Kolligs J Siefer OG Upregulation of AKR1C1 and AKR1C3 expression in OPSCC with integrated HPV16 and HPV-negative tumors is an indicator of poor prognosis. Int J Cancer (2019) 144:2465–77. 10.1002/ijc.31954 30367463

[108] Attaran N Coates PJ Zborayova K Sgaramella N Nylander K Gu X . Upregulation of apoptosis related genes in clinically normal tongue contralateral to squamous cell carcinoma of the oral tongue, an effort to maintain tissue homeostasis. Head Neck Pathol (2024) 18:89. 10.1007/s12105-024-01695-6 39348078 PMC11442960

[109] Horn D Freudlsperger C Holzinger D Kunzmann K Plinkert P Dyckhoff G Upregulation of pAKT(Ser473) expression in progression of HPV-positive oropharyngeal squamous cell carcinoma. Head Neck (2017) 39:2397–405. 10.1002/hed.24910 28945300

[110] Baddevithana AK Jayasinghe RD Tilakaratne WM Illeperuma RP Siriwardena BSMS . Expression of human papillomavirus and the P16 gene in oral potentially malignant disorders (OPMD): a comparative study with oral squamous cell carcinoma. Appl Immunohistochem Mol Morphol (2023) 31:331–8. 10.1097/PAI.0000000000001124 37036407

[111] Pflumio C Thomas J Salleron J Faivre J-C Borel C Dolivet G Expression of immune response biomarkers (PD-L1, P16, CD3+ and CD8+ TILs) in recurrent head and neck squamous cell carcinoma within previously irradiated areas. Oncol Rep (2021) 45:1273–83. 10.3892/or.2021.7928 33432367

[112] Ibrahim SO Bertelsen B Kalvenes MB Idris AM Vasstrand EN Nilsen R Expression of keratin 13, 14 and 19 in oral squamous cell carcinomas from Sudanese snuff dippers: lack of association with human papillomavirus infection. APMIS (1998) 106:959–69. 10.1111/j.1699-0463.1998.tb00246.x 9833698

[113] Nasry WHS Jones K Rodriguez-Lecompte JC Tesch M Martin CK . Expression of mPGES1 and P16 in feline and human oral squamous cell carcinoma: a comparative oncology approach. Vet Comp Oncol (2024) 22:204–16. 10.1111/vco.12967 38378135

[114] Fregonezi PAG Silva TGA Simões RT Moreau P Carosella ED Kläy CPM Expression of nonclassical molecule human leukocyte Antigen-G in oral lesions. Am J Otolaryngol (2012) 33:193–8. 10.1016/j.amjoto.2010.08.001 21035918

[115] Pandiar D Nayanar SK Babu S Babu S . Expression of P16(INK4a) in oropharyngeal squamous cell carcinoma from a tertiary cancer centre of South India: a preliminary study. Indian J Med Res (2021) 154:497–503. 10.4103/ijmr.IJMR_386_19 35345076 PMC9131790

[116] Nemes JA Deli L Nemes Z Márton IJ . Expression of P16(INK4A), P53, and Rb proteins are independent from the presence of human papillomavirus genes in oral squamous cell carcinoma. Oral Surg Oral Med Oral Pathol Oral Radiol Endod (2006) 102:344–52. 10.1016/j.tripleo.2005.10.069 16920543

[117] Inchingolo AD Dipalma G Inchingolo AM Malcangi G Santacroce L D’Oria MT The 15-Months clinical experience of SARS-CoV-2: a literature review of therapies and adjuvants. Antioxidants (Basel) (2021) 10:881. 10.3390/antiox10060881 34072708 PMC8226610

[118] Santacroce L Charitos IA Ballini A Inchingolo F Luperto P De Nitto E The human respiratory system and its microbiome at a glimpse. Biology (Basel) (2020) 9:318. 10.3390/biology9100318 33019595 PMC7599718

[119] Inchingolo F Inchingolo AM Piras F Ferrante L Mancini A Palermo A The interaction between gut microbiome and bone health. Curr Opin Endocrinol Diabetes Obes (2024) 31:122–30. 10.1097/MED.0000000000000863 38587099 PMC11062616

[120] Balzanelli MG Rastmanesh R Distratis P Lazzaro R Inchingolo F Del Prete R The role of SARS-CoV-2 spike protein in long-term damage of tissues and organs, the underestimated role of retrotransposons and stem cells, a working hypothesis. Endocr Metab Immune Disord Drug Targets (2025) 25:85–98. 10.2174/0118715303283480240227113401 38468535

[121] Mancini A Inchingolo AM Marinelli G Trilli I Sardano R Pezzolla C Topical and systemic therapeutic approaches in the treatment of oral Herpes simplex virus infection: a systematic review. Int J Mol Sci (2025) 26:8490. 10.3390/ijms26178490 40943411 PMC12428954

[122] Tezal M Scannapieco FA Wactawski-Wende J Hyland A Marshall JR Rigual NR Local inflammation and human papillomavirus status of head and neck cancers. Arch Otolaryngol Head Neck Surg (2012) 138:669–75. 10.1001/archoto.2012.873 22710409 PMC4477511

[123] Saleh W Cha S Banasser A Fitzpatrick SG Bhattacharyya I Youssef JM Localization and characterization of human Papillomavirus-16 in oral squamous cell carcinoma. Oral Dis (2023) 29:436–44. 10.1111/odi.13920 34022097

[124] Bentzen J Toustrup K Eriksen JG Primdahl H Andersen LJ Overgaard J . Locally advanced head and neck cancer treated with accelerated radiotherapy, the hypoxic modifier nimorazole and weekly cisplatin. Results from the DAHANCA 18 phase II study. Acta Oncol (2015) 54:1001–7. 10.3109/0284186X.2014.992547 25629651

[125] Contrera KJ Smile TD Mahomva C Wei W Adelstein DJ Broughman JR Locoregional and distant recurrence for HPV-associated oropharyngeal cancer using AJCC 8 staging. Oral Oncol (2020) 111:105030. 10.1016/j.oraloncology.2020.105030 33038751

[126] Sobti A Sharif-Askari FS Khan S Sharif-Askari NS Hachim MY Williams L Logistic regression prediction model identify type 2 diabetes mellitus as a prognostic factor for human Papillomavirus-16 associated head and neck squamous cell carcinoma. PLoS One (2019) 14:e0217000. 10.1371/journal.pone.0217000 31095649 PMC6522118

[127] Shah A Malik A Garg A Mair M Nair S Chaturvedi P . Oral sex and human papilloma virus-related head and neck squamous cell cancer: a review of the literature. Postgrad Med J (2017) 93:704–9. 10.1136/postgradmedj-2016-134603 28778951

[128] Huang L-W Seow K-M . Oral sex is a risk factor for human papillomavirus-associated nasopharyngeal carcinoma in husbands of women with cervical cancer. Gynecol Obstet Invest (2010) 70:73–5. 10.1159/000291199 20215764

[129] Muscatello LV Avallone G Benazzi C Sarli G Porcellato I Brachelente C Oral squamomelanocytic tumour in a dog: a unique biphasic cancer. J Comp Pathol (2016) 154:211–4. 10.1016/j.jcpa.2015.12.004 26805740

[130] Fracchioli S Porpiglia M Arisio R Voglino G Katsaros D . Oral squamous carcinoma in a patient with cervix cancer: use of human papillomavirus analysis to differentiate synchronous *versus* metastatic tumor. Gynecol Oncol (2003) 89:522–5. 10.1016/s0090-8258(03)00129-x 12798722

[131] Cohan DM Popat S Kaplan SE Rigual N Loree T Hicks WLJ . Oropharyngeal cancer: current understanding and management. Curr Opin Otolaryngol Head Neck Surg (2009) 17:88–94. 10.1097/moo.0b013e32832984c0 19373958

[132] Fakhry C Andersen KK Eisele DW Gillison ML . Oropharyngeal cancer survivorship in Denmark, 1977-2012. Oral Oncol (2015) 51:982–4. 10.1016/j.oraloncology.2015.08.006 26319353

[133] Brown LM Check DP Devesa SS . Oropharyngeal cancer incidence trends: diminishing racial disparities. Cancer Causes Control (2011) 22:753–63. 10.1007/s10552-011-9748-1 21380619

[134] Stojanov IJ Woo S-B . Human papillomavirus and epstein-barr virus associated conditions of the oral mucosa. Semin Diagn Pathol (2015) 32:3–11. 10.1053/j.semdp.2014.12.003 25749203

[135] Atula S Auvinen E Grenman R Syrjänen S . Human papillomavirus and epstein-barr virus in epithelial carcinomas of the head and neck region. Anticancer Res (1997) 17:4427–33. 9494545

[136] Nair S Pillai MR . Human papillomavirus and disease mechanisms: relevance to oral and cervical cancers. Oral Dis (2005) 11:350–9. 10.1111/j.1601-0825.2005.01127.x 16269025

[137] Saulle R Semyonov L Mannocci A Careri A Saburri F Ottolenghi L Human papillomavirus and cancerous diseases of the head and neck: a systematic review and meta-analysis. Oral Dis (2015) 21:417–31. 10.1111/odi.12269 24962169

[138] Gonzalez-Losa Mdel R Canul-Canche J Calderon-Rocher C . Human papillomavirus 58 in a squamous cell carcinoma of the tongue. Oral Oncol (2009) 45:e72. 10.1016/j.oraloncology.2009.02.002 19362048

[139] Coscia MF Monno R Ballini A Mirgaldi R Dipalma G Pettini F Human papilloma virus (HPV) genotypes prevalence in a region of south Italy (apulia). Ann Ist Super Sanita (2015) 51:248–51. 10.4415/ANN_15_03_14 26428051

[140] Xu MJ Plonowska KA Gurman ZR Humphrey AK Ha PK Wang SJ Treatment modality impact on quality of life for human papillomavirus-associated oropharynx cancer. Laryngoscope (2020) 130:E48–E56. 10.1002/lary.27937 30919470

[141] Manley C Hutchinson C Mahajan A Ibrahim O Folch E Kumar R . Treatment of recurrent respiratory papillomatosis: case series and review of technique. Surg Technol Int (2021) 38:139–43. 10.52198/21.STI.38.GS1408 33844241

[142] Meccariello G Catalano A Cammaroto G Iannella G Vicini C Hao S-P Treatment options in early stage (stage I and II) of oropharyngeal cancer: a narrative review. Medicina (Kaunas) (2022) 58:1050. 10.3390/medicina58081050 36013517 PMC9415053

[143] Nagel R Martens-de Kemp SR Buijze M Jacobs G Braakhuis BJM Brakenhoff RH . Treatment response of HPV-positive and HPV-negative head and neck squamous cell carcinoma cell lines. Oral Oncol (2013) 49:560–6. 10.1016/j.oraloncology.2013.03.446 23578372

[144] Pitak-Arnnop P Witohendro L-K Meningaud J-P Subbalekha K Iamaroon A Sirintawat N Which characteristics can be expected from P16(+)-Squamous cell carcinomas of the posterior oral cavity and oropharynx? - distinctive results from central Germany. J Stomatol Oral Maxillofac Surg (2020) 121:213–8. 10.1016/j.jormas.2019.10.013 31676425

[145] Cipriani NA Blair E Taxy JB . WHIM syndrome and oral squamous cell carcinoma. Oral Surg Oral Med Oral Pathol Oral Radiol Endod (2010) 109:105–8. 10.1016/j.tripleo.2009.08.011 19926501

[146] Qin T Li S Henry LE Chou E Cavalcante RG Garb BF Whole-genome CpG-Resolution DNA methylation profiling of HNSCC reveals distinct mechanisms of carcinogenesis for fine-scale HPV+ cancer subtypes. Cancer Res Commun (2023) 3:1701–15. 10.1158/2767-9764.CRC-23-0009 37654626 PMC10467604

[147] Caicedo-Granados E Lin R Fujisawa C Yueh B Sangwan V Saluja A . Wild-type P53 reactivation by small-molecule Minnelide^TM^ in human papillomavirus (HPV)-positive head and neck squamous cell carcinoma. Oral Oncol (2014) 50:1149–56. 10.1016/j.oraloncology.2014.09.013 25311433 PMC4678773

[148] Inchingolo R Acquafredda F Tedeschi M Laera L Surico G Surgo A Worldwide management of hepatocellular carcinoma during the COVID-19 pandemic. World J Gastroenterol (2021) 27:3780–9. 10.3748/wjg.v27.i25.3780 34321843 PMC8291009

[149] Scarano A Inchingolo F Scogna G Leo L Crisante A Greco Lucchina A Xanthelasma palpebrarum removed with atmospheric plasma technique: 11-year follow up. J Biol Regul Homeost Agents (2021) 35:181–5. 10.23812/21-2supp1-18 34281315

[150] Inchingolo F Inchingolo AD Riccaldo L Costa S Palermo A Inchingolo AM Weight and dental eruption: the correlation between BMI and eruption. Eur J Paediatr Dent (2025) 1:298–305. 10.23804/ejpd.2025.2220 40266019

[151] Inchingolo AM Malcangi G Ferrante L Del Vecchio G Viapiano F Inchingolo AD Surface coatings of dental implants: a review. J Funct Biomater (2023) 14:287. 10.3390/jfb14050287 37233397 PMC10218820

[152] Sgaramella N Coates PJ Strindlund K Loljung L Colella G Laurell G Expression of P16 in squamous cell carcinoma of the Mobile tongue is independent of HPV infection despite presence of the HPV-receptor Syndecan-1. Br J Cancer (2015) 113:321–6. 10.1038/bjc.2015.207 26057450 PMC4506391

[153] Chen X-J Sun K Jiang W-W . Absence of high-risk HPV 16 and 18 in Chinese patients with oral squamous cell carcinoma and oral potentially malignant disorders. Virol J (2016) 13:81. 10.1186/s12985-016-0526-2 27206495 PMC4875721

[154] de Abreu PM Có ACG Azevedo PL do Valle IB de Oliveira KG Gouvea SA Frequency of HPV in oral cavity squamous cell carcinoma. BMC Cancer (2018) 18:324. 10.1186/s12885-018-4247-3 29580212 PMC5870524

[155] Nopmaneepaisarn T Tangjaturonrasme N Rawangban W Vinayanuwattikun C Keelawat S Bychkov A . Low prevalence of P16-Positive HPV-related head-neck cancers in Thailand: tertiary referral center experience. BMC Cancer (2019) 19:1050. 10.1186/s12885-019-6266-0 31694600 PMC6836494

[156] Tokuzen N Nakashiro K-I Tojo S Goda H Kuribayashi N Uchida D . Human Papillomavirus-16 infection and P16 expression in oral squamous cell carcinoma. Oncol Lett (2021) 22:528. 10.3892/ol.2021.12789 34055093 PMC8138897

[157] Li P Fang Q Yang Y Chen D Du W Liu F Survival significance of number of positive lymph nodes in oral squamous cell carcinoma stratified by P16. Front Oncol (2021) 11:545433. 10.3389/fonc.2021.545433 33747901 PMC7969991

[158] Sri S Ramani P Premkumar P Ramshankar V Ramasubramanian A Krishnan RP . Prevalence of human papillomavirus (HPV) 16 and 18 in oral malignant and potentially malignant disorders: a polymerase chain reaction analysis – a comparative study. Ann Maxillofac Surg (2021) 11:6–11. 10.4103/ams.ams_376_20 34522646 PMC8407623

[159] Silveira HA Almeida LY Carlos R Silva EV Ferrisse TM Duarte A Human papillomavirus Co-Infection and survival in oral and oropharyngeal squamous cell carcinoma: a study in 235 Brazilian patients. Auris Nasus Larynx (2022) 49:258–70. 10.1016/j.anl.2021.06.006 34274177

[160] Petrović A Čanković M Avramov M Popović ŽD Janković S Mojsilović S . High-risk human papillomavirus in patients with oral carcinoma and oral potentially malignant disorders in Serbia-A pilot study. Medicina (Kaunas) (2023) 59:1843. 10.3390/medicina59101843 37893561 PMC10608774

[161] Tangthongkum M Phisalmongkhon S Leelasawatsuk P Supanimitjaroenporn P Kirtsreesakul V Tantipisit J . Impact of human papillomavirus status on survival in patients with oral cancer. Laryngoscope Investig Otolaryngol (2024) 9:e1294. 10.1002/lio2.1294 38867852 PMC11168070

[162] Becker A-S Merkel J Bozkurt I Strüder DF Maletzki C Hühns M P16 overexpression identifies oncogenic high-risk HPV infection in non-oropharyngeal squamous cell carcinoma of the head and neck. Head and Neck (2024) 46:2569–81. 10.1002/hed.27764 38594829

